# West Nile virus transmission: results from the integrated surveillance system in Italy, 2008 to 2015

**DOI:** 10.2807/1560-7917.ES.2016.21.37.30340

**Published:** 2016-09-15

**Authors:** Caterina Rizzo, Christian Napoli, Giulietta Venturi, Simonetta Pupella, Letizia Lombardini, Paolo Calistri, Federica Monaco, Roberto Cagarelli, Paola Angelini, Romeo Bellini, Marco Tamba, Alessandra Piatti, Francesca Russo, Giorgio Palù, Mario Chiari, Antonio Lavazza, Antonino Bella, the Italian WNV surveillance working group

**Affiliations:** 1 National Institute of Health (Istituto Superiore di Sanità, ISS), Italy; 2 National Blood Centre, National Institute of Health (Istituto Superiore di Sanità, ISS), Italy; 3 National Transplant Centre, National Institute of Health (Istituto Superiore di Sanità, ISS), Italy; 4 Istituto Zooprofilattico Sperimentale dell’Abruzzo e del Molise “G. Caporale”, Italy; 5 Regional Health Authority of Emilia-Romagna, Italy; 6 Centro Agricoltura Ambiente “G. Nicoli”, Crevalcore, Italy; 7 Istituto Zooprofilattico Sperimentale della Lombardia e dell’Emilia-Romagna, Brescia, Italy; 8 Regional Health Authority of Lombardy, Italy; 9 Regional Health Authority of Veneto, Italy; 10 Department of Molecular Medicine, University of Padua, Padua, Italy; 11 Istituto Zooprofilattico Sperimentale della Lombardia e dell’Emilia-Romagna ‘Bruno Ubertini’, Brescia, Italy; 12 Members of the group are listed at the end of the article

**Keywords:** West Nile virus, integrated surveillance, Italy, incidence

## Abstract

In Italy a national Plan for the surveillance of imported and autochthonous human vector-borne diseases (chikungunya, dengue, Zika virus disease and West Nile virus (WNV) disease) that integrates human and veterinary (animals and vectors) surveillance, is issued and revised annually according with the observed epidemiological changes. Here we describe results of the WNV integrated veterinary and human surveillance systems in Italy from 2008 to 2015. A real time data exchange protocol is in place between the surveillance systems to rapidly identify occurrence of human and animal cases and to define and update the map of affected areas i.e. provinces during the vector activity period from June to October. WNV continues to cause severe illnesses in Italy during every transmission season, albeit cases are sporadic and the epidemiology varies by virus lineage and geographic area. The integration of surveillance activities and a multidisciplinary approach made it possible and have been fundamental in supporting implementation of and/or strengthening preventive measures aimed at reducing the risk of transmission of WNV trough blood, tissues and organ donation and to implementing further measures for vector control.

## Introduction

West Nile virus (WNV), a single stranded RNA virus of the genus Flavivirus, is mostly transmitted by mosquito bites, but also through organ transplantation, blood transfusion, in the laboratory setting and from mother to child during pregnancy, delivery, or breastfeeding [1]. The virus is maintained in a continuous vertebrate-mosquito cycle. Mosquitoes are the vectors and birds are the reservoir for West Nile virus. Humans, horses and other mammals are considered dead-end hosts and do not contribute to further spread of the disease.

In humans, ca 80% of infections are asymptomatic, 20% of those infected may present with fever and or influenza-like symptoms, whereas less than 1% i.e. mostly the elderly and immunocompromised people, develop West Nile neuroinvasive diseases (WNND) such as encephalitis, meningo-encephalitis or meningitis that may lead to death [1,2].

In 1996, the first large human outbreak of WND in Europe was reported in Romania with 393 confirmed cases [3] and since then, the number of WNND cases reported in humans increased significantly. From 2002 to 2009, several WNV outbreaks were reported in a few European and neighbouring countries (Albania, Bosnia, Bulgaria, Croatia, FYROM, Greece, Hungary, Italy, Kosovo, Montenegro, Portugal, Romania, Russia, Serbia, Spain, Turkey, Ukraine) [4]. After 2005, an endemic transmission cycle started in some south-eastern and eastern European countries with annual outbreaks [5-8], mostly sustained by the rapid spread of WNV lineage 2 strains belonging to the Hungarian and Volgograd clade [9].

In Italy, the first outbreak of WNV infection was reported in 1998 in the region of Tuscany [10] and since 2001, a national veterinary surveillance plan for WNV based on wild bird mortality and on entomological and sentinel animal surveillance has been in place. The plan aims at monitoring areas at risk for WNV circulation, and detecting WNV seroconversion in horses in these areas [10,11]. In parallel, in 2002, human surveillance recommendations were issued by the Ministry of Health requesting all 21 Italian regions and autonomous provinces and autonomous provinces to report to the national mandatory surveillance system all hospitalised cases of aseptic meningitis and encephalitis with unknown aetiology, and cases of fever with rash in the areas where veterinary cases where identified. Moreover, health authorities were requested to actively identify cases and possible WNV seroconversion in close contacts of infected animals such as employees of stables and veterinarians or people living in the area [12].

The veterinary and human surveillance systems did not detect any relevant circulation of WNV until 2008, when the virus was identified in mosquitoes, birds, horses and humans in the area surrounding the Po river delta, involving three north Italian regions [2].

Since the re-introduction of the virus in 2008, a constant and intensified WNV circulation across the whole of Italy was observed with a geographical spread of WNV to the west and south [2,13]. Moreover, from 2008 to 2011, WNV lineage 1 was responsible for reported human WNDD cases, but, since 2011, evidence of extensive circulation of lineage 2 closely related to both the Hungarian and Volgograd clades, was demonstrated [13-15]. This suggests a possible introduction of lineage 2 from central and/or eastern European countries, probably through migratory birds [16,17].

WNV has caused severe illnesses in humans in Italy every season over nearly a decade. However, cases occur sporadically and the epidemiology varies according to the virus lineage and the affected geographic area. Integrated surveillance is essential to identify outbreaks in a timely fashion and to guide prevention efforts aimed at reducing the incidence of severe cases and at reducing the probability of virus transmission via blood, tissue and organ donations.

Here we present the evolution of the national surveillance plan in the five years following its first implementation, and briefly describe results of the WNV integrated surveillance system in Italy.

## Methods

### The integrated veterinary and human surveillance systems in Italy

In 2008 and 2011, the national WNV veterinary surveillance plan [18] and the WNND human surveillance recommendations [19] were revised, respectively. New activities were added including the potential integration between veterinary (animals and vectors) and human surveillance. Provinces with evidence of animal and vector or human infections in the previous season have to implement active surveillance and mandatory screening of blood donations. The veterinary and human plans have been published separately and they were revised annually according to the epidemiological situation in the country. Since 2009, some north Italian regions have implemented an integrated surveillance, targeting mosquitoes, birds, and humans [15].

Pillars of the national integrated surveillance system are (i) the entomological monitoring based on mosquito collections in selected sites; (ii) the animal surveillance targeting migratory and resident birds as well as horses and poultry; (iii) the human surveillance system requesting clinicians to report all possible, probable and confirmed WNV cases, irrespective of age, using a modified European case definition which includes neurological symptoms in the clinical criteria [2].

#### Veterinary surveillance

For veterinary surveillance purposes, the Italian territory was subdivided in two distinct epidemiological territories: endemic and non-endemic. The former includes the territories where WNV was detected in the previous two years. At present these are the plain of the Po river valley, including Friuli Venezia Giulia, Emilia Romagna, Lombardy, Piedmont, Veneto regions, and the two main Italian islands: Sardinia and Sicily. The remainder of Italy is considered non-endemic. In the endemic territories, an early warning system is in place, which enables the reinforcement of the activities aiming at detecting WNV in vectors and birds [20]. In particular, the regions of the plain of the Po river i.e. Friuli Venezia Giulia, Emilia Romagna, Lombardy, Piedmont, Veneto, operate an enhanced surveillance system based on a network of fixed mosquito traps (in grids from 10 to 20 km) and on the collection of residential wild birds, mainly *Corvidae.* Timely data on viral circulation triggers preventive measures to avoid the virus transmission via blood, tissue and organ donations [15,21,22].

Data collected through the Veterinary Plan are registered using an information system (SISMAN) that records and manages laboratory results and publishes weekly and daily reports describing the outcomes of the surveillance activities. A web-based geographic information system (WebGIS) was developed for displaying thematic maps and to help the veterinary services to explore the area surrounding the outbreak, and to create buffers around the reported cases.

#### Human surveillance

Human cases are notified by regional and local authorities to the Ministry of Health and to the Istituto Superiore di Sanità (ISS, national public health institute) using a specific password-protected web-based system, which permits to report probable and confirmed cases, adding available epidemiological (including the province of exposure), clinical and laboratory information. The web-based system is accessible also to the National Blood Center (NBC) and to the National Transplant Center (NTC), which implement precautionary measures on blood donation and transplant activities also on the basis of data on WNV human cases.

In order to rapidly identify, define and update the map of the affected areas i.e. provinces during the vector activity period, a real-time data exchange protocol is in place between the two systems.

### Definitions and identification of at risk areas in Italy

The national plan for human surveillance defines as ‘affected areas’ all the provinces (Nomenclature of Units for Territorial Statistics (NUTS)-3) [23] where laboratory-confirmed WNV infections in animals, vectors or humans, irrespective of age, were notified in the previous years or during the surveillance period. The ‘surveillance period’ covers the months between the 1 June and 30 October, which is considered the period with the highest vector activity. Identification of an affected area immediately triggers the definition of the ‘surveillance area’ for the whole region (NUTS-2 level) where the affected area is located. In the surveillance area, passive human surveillance has to be set up, and physicians are requested to report all possible, probable and confirmed WNND cases. In the affected areas, the NBC and the NTC immediately activate the WNV screening, by Nucleic Acid Amplification Test (NAT), of all blood, blood component, and organ donations until the end of the vector season in order to avoid WNV transmission [24].

### Data analysis

In our analysis we included autochthonous confirmed cases of WNND, West Nile fever and infections detected in blood and in organ donors; we also analysed data on vectors and birds. Annual incidence rates in human in the period from 2008 to 2015 were calculated using annual resident province population. All population data were obtained from the Italian National Institute of Statistics (ISTAT) [8]. The statistical analysis was carried out using STATA software version 11.2 (Stata Corporation, College Station, TX, US). Maps were produced using Epi Info version 7 (CDC, Atlanta, GA, US).

## Results

**Figure 1:**
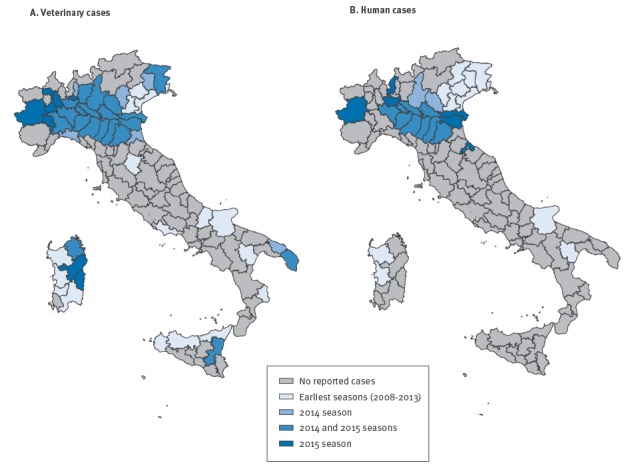
Geographical distribution of West Nile neuroinvasive disease in horses (panel A) and humans (panel B), Italy 2008–2015

In Italy from 2008 to 2015, the circulation of WNV was reported in mosquitoes, birds and horses in the territory of 14 regions, with 173 indigenous cases of human WNND notified in eight regions (Apulia, Basilicata, Emilia-Romagna, Friuli Venezia Giulia, Lombardy, Piedmont, Sardinia, Veneto). Figure 1 shows the geographical distribution of human and equine neuroinvasive cases detected in Italy from 2008 to 2015.

Results of the veterinary surveillance from 2008 to 2015 are reported in Table 1. Since 2008, more than 16,000 mosquito pools have been tested, with 435 positive results, in eight regions and 30 provinces where subsequently human WNND cases were reported. Positive mosquito pools without consecutive or previous detection of WNND in humans were detected in 2008 in the province of Brescia, Lombardy region (n=1); in 2011 in the province of Messina, Sicily region (n=1) and in 2014 in the province of Genoa province, Liguria region and in the province of Alessandria province, Piedmont region (n=2, respectively). 

**Table 1:** Veterinary surveillance for West Nile virus in birds, mosquitoes, horses and chickens, Italy by year, 2008–2015

Years	Mosquitoes pool		Birds		Horses		Chickens	
	Tested	Positive	Tested	Positive	Tested	Positive	Tested	Positive
**2008**	152	8	490	45	1,532	563	76	1
**2009**	217	20	3,753	22	4,430	223	2,517	0
**2010**	1,068	13	4,182	3	2,728	128	1,213	5
**2011**	2,113	8	3,026	11	3,424	197	2,505	34
**2012**	2,366	14	2,260	21	2,081	63	2,461	6
**2013**	2,324	146	3,761	79	2,735	50	2,363	0
**2014**	5,834	125	5,368	48	5,882	27	1,974	7
**2015**	2,300	101	2,147	67	1,313	30	24	1
**Total**	**16,374**	**435**	**24,987**	**296**	**24,125**	**1,281**	**13,133**	**54**

**Figure 2:**
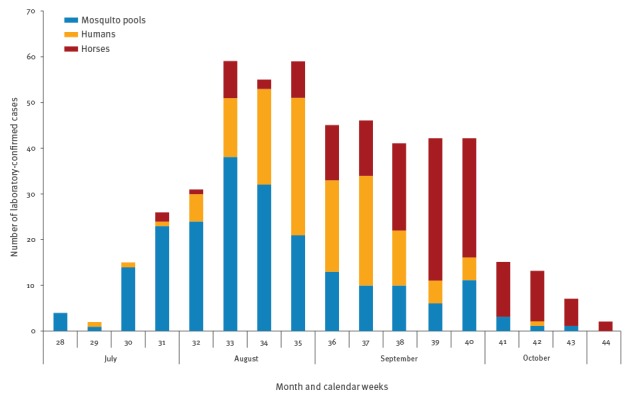
West Nile virus detections in the veterinary and human surveillance by month, Italy, 2008–2015

In the provinces where there were also human cases, viral circulation in mosquitoes preceded human WNV (WNND and West Nile fever) cases with a mean of 22 days (range 0–58), except for few provinces (2011: n=1, 2012: n=2, 2013 and 2015: n=3, respectively) where the identification of human cases anticipated the evidence of viral circulation in vectors (Figure 2).

Also from 2008 to 2015, more than 24,000 residential birds were examined, with 296 positive results. WNV was found in birds in six regions. In the 15 provinces where there were human cases, positive birds were identified with a mean of 35 days (range 1–315 days) before the appearance of human cases.

During 2008 to 2010, all isolated viruses belonged to WNV-lineage 1, from 2011 to 2012 lineage 1 and 2 co-circulated with a higher proportion of lineage 1 and from 2013 to 2015 lineage 2 prevailed over lineage 1 with evidence of lineage 1 circulation only in one province each season.

### Human cases

The national incidence of WNND peaked in 2013 (1.66/1,000,0000 inhabitants) and 2015 (1.34/1,000,0000). From 2008 to 2011, the annual incidence was relatively low (median: 0.41/1,000,000; range: 0.11–0.64). From 2012 to 2015, the national incidence of WNND increased by threefold (median: 1.20/1,000,0000; range: 0.78–1.66). The increase in disease incidence was initially concentrated in northern Italy moving over time towards the south, mainly to areas in central and to some areas in southern Italy. However, 91% (157/173) of the WNND cases detected during the entire study period were reported from three regions (Emilia-Romagna, Lombardy and Veneto) in the Po river plain area (Table 2) with the Emilia-Romagna and Veneto regions reporting the highest incidence (1.60 and 1.46/1,000,000 respectively).

**Table 2:** Incidence of human West Nile neuroinvasive disease cases per 1,000,000 inhabitants by province and year, Italy 2008–2015 (n=173)

Region	Province	2008		2009		2010		2011		2012		2013		2014		2015	
		*N.*	*Incidence*	*N.*	*Incidence*	*N.*	*Incidence*	*N.*	*Incidence*	*N.*	*Incidence*	*N.*	*Incidence*	*N.*	*Incidence*	*N.*	*Incidence*
Piedmont	Torino	0	NA	0	NA	0	NA	0	NA	0	NA	0	NA	0	NA	1	0.44
Lombardy	Brescia	0	NA	0	NA	0	NA	0	NA	0	NA	2	1.60	1	0.79	0	NA
	Cremona	0	NA	0	NA	0	NA	0	NA	0	NA	1	2.76	3	8.28	4	11.06
	Lodi	0	NA	0	NA	0	NA	0	NA	0	NA	1	4.43	2	8.73	3	13.07
	Mantova	0	NA	2	4.88	0	NA	0	NA	0	NA	6	14.59	2	4.82	3	7.23
	Milano	0	NA	0	NA	0	NA	0	NA	0	NA	0	NA	0	NA	4	1.25
	Pavia	0	NA	0	NA	0	NA	0	NA	0	NA	0	NA	5	9.12	5	9.11
Veneto	Belluno	0	NA	0	NA	0	NA	1	4.68	0	NA	0	NA	0	NA	0	NA
	Padova	0	NA	0	NA	0	NA	0	NA	0	NA	1	1.08	0	NA	0	NA
	Rovigo	3	12.18	5	20.23	0	NA	0	NA	0	NA	5	20.61	0	NA	1	4.12
	Treviso	0	NA	0	NA	0	NA	6	6.75	6	6.85	4	4.54	0	NA	0	NA
	Venezia	1	1.18	1	1.17	2	2.33	1	1.16	15	17.72	2	2.36	0	NA	0	NA
	Verona	0	NA	0	NA	0	NA	0	NA	0	NA	1	1.10	1	1.08	0	NA
	Vicenza	1	1.17	0	NA	1	1.15	0	NA	0	NA	0	NA	0	NA	0	NA
Friuli Venezia Giulia	Gorizia	0	NA	0	NA	0	NA	0	NA	1	7.15	0	NA	0	NA	0	NA
	Pordenone	0	NA	0	NA	0	NA	0	NA	2	6.44	0	NA	0	NA	0	NA
	Udine	0	NA	0	NA	0	NA	2	3.69	1	1.87	0	NA	0	NA	0	NA
Emilia-Romagna	Bologna	1	1.04	2	2.05	0	NA	0	NA	0	NA	1	1.01	1	1.00	2	1.99
	Ferrara	2	5.62	5	13.97	0	NA	0	NA	0	NA	5	14.18	0	NA	1	2.82
	Modena	0	NA	2	2.91	0	NA	0	NA	0	NA	7	10.17	2	2.85	8	11.39
	Parma	0	NA	0	NA	0	NA	0	NA	0	NA	1	2.32	1	2.26	3	6.74
	Piacenza	0	NA	0	NA	0	NA	0	NA	0	NA	0	NA	2	6.93	1	3.47
	Reggio nell'Emilia	0	NA	0	NA	0	NA	0	NA	0	NA	6	11.48	1	1.87	1	1.88
	Rimini	0	NA	0	NA	0	NA	0	NA	0	NA	0	NA	0	NA	1	2.98
Apulia	Foggia	0	NA	0	NA	0	NA	0	NA	0	NA	1	1.59	0	NA	0	NA
Basilicata	Matera	0	NA	0	NA	0	NA	0	NA	1	5.00	0	NA	0	NA	0	NA
Sardinia	Oristano	0	NA	0	NA	0	NA	3	18.05	2	12.22	0	NA	0	NA	0	NA
	Sassari	0	NA	0	NA	0	NA	1	2.97	0	NA	0	NA	0	NA	0	NA

NA: not applicable.

**Figure 3:**
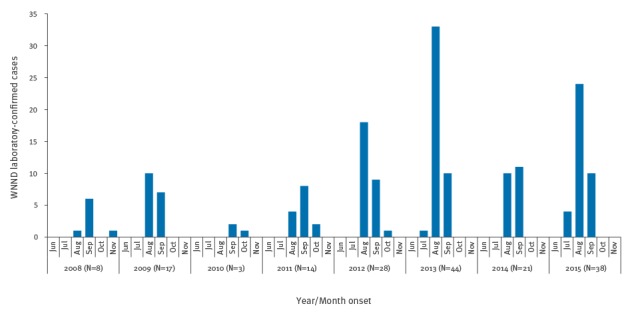
Human West Nile neuroinvasive disease cases by month of symptom onset and year, Italy 2008–2015 (n=173)

The distribution of human WNND cases by month and year of symptom onset is reported in Figure 3.

WNND: West Nile neuroinvasive diseases.

The median age of cases was 73 years (range: 10–90 years) during the entire surveillance period, varying from a minimum of 67 years (range: 41–68 years) in 2010 to 77 years (range: 42–89 years) in 2013. Most of the reported cases (69%, 120/173) were male. All WNND cases were hospitalised: 82 presented as encephalitis, 44 as meningo-encephalitis, 31 as meningitis, seven as polyradiculoneuritis, one as facial paralysis and eight as other neurological symptoms: meningeal symptoms (n=2), extrapyramidal syndrome (n=1), confusion (n=1), headache (n=1), ataxic paraparesis (n=1), neuropathy of lower limbs (n=1), symptoms were not specified for one case. Eighteen of the 173 WNND cases died (2009: n=3; 2011: n=5; 2012: n=1; 2013: n=7; 2014 and 2015: n=1 each), corresponding to an overall case fatality rate of 10%. The median age of WNND fatal cases was 82 years (range: 34–89 years), 14 of 18 were male. All fatal cases were reported to have chronic conditions before symptom onset and 10 presented with encephalitis, six with meningo-encephalitis and two with meningitis.

In the entire period, 69 confirmed cases of WNV fever were reported to the national surveillance system from four Italian regions (Emilia-Romagna n=31, Lombardy n=3, Marche n=1, and Veneto n=34).

### Situation in 2015

In Italy in 2015, since the beginning of the surveillance period, for the first time since 2008, four cases, two from Emilia-Romagna and Lombardy region, respectively, all had symptom onset in the last week of July (calendar week 31). The last human cases reported had onset of symptoms or were detected in mid-September and the integrated surveillance detected signals of WNV circulation (veterinary cases), for the first time ever, in a northern-western region (Piedmont), where also one WNND case was reported. Moreover, virus circulation was detected in animals for the first time in the southern Italian regions Apulia (1 horse) and Sicily (1 chicken), where no human cases were reported. 

In 2015, a total of 316,614 WNV NAT screening tests were conducted in blood donors in the affected provinces and 13 asymptomatic donors, six in Emilia Romagna and seven in Lombardy were identified. No donor or organ transplant recipients were positive for WNV among the 168 tested.

## Discussion and conclusions

In Italy from 2008 to 2015, the circulation of WNV was reported in mosquitoes, birds and horses in the territory of 14 regions [10], with 173 auchtotonous cases of human WNND notified. [11]. A peak of cases was reported in 2013, a second peak was observed in 2015. From 2008 to 2015, the Italian contribution to the European case load increased substantially i.e. from a minimum of 11% (14/128) in 2011 to a maximum of 56% (60/108) in 2015 [3,9]. Moreover, in this period, an expansion of the Italian affected areas from the north-east to the north-west and south was observed.

From 2008 to 2010, only circulation of lineage 1 was reported. The complexity of the epidemiological scenario increased in 2011 with the detection of the novel lineage 2 which overcame lineage 1 from 2013 to 2015 and was responsible for both human and animal cases [12]. It is not clear why there was more WNV activity in the past 4 years of surveillance (2012–2015) compared to the earlier surveillance period (2008–2011). However, virus lineage and virulence, weather conditions, bird population size and immunity, vector density, and human behaviour, are all factors that play a role in determining if, when and where human outbreaks may occur.

In Europe, since 2008, WNV has spread into areas not previously affected, including Greece [25], Portugal [26], Turkey [27], and many eastern European countries (Albania, Bosnia and Herzegovina, Bulgaria, Croatia, the former Yugoslav Republic of Macedonia, Kosovo under UN Security Council Resolution 1244, Montenegro, Serbia) [28-30]. In the same period the disease has been reported in Hungary, Israel, Italy, Romania, Russia, Spain and Ukraine [2,29]. 

The fact that the WNV has become endemic in Italy has brought the local and national Italian authorities to strengthen the WNV surveillance system and for this reason, probably, Italy is the country with the highest number of reported cases in EU, while other EU countries have different epidemiological situations, with different surveillance systems and objectives [31].

Surveillance of WNV circulation requires an interdisciplinary approach given the complexity of the viral biological cycle. For this reason, the integration of entomological, veterinary and human surveillance systems is an essential tool for public health. In fact, the veterinary and entomological surveillance activities are crucial for estimating the public health risk associated with WNV, and for the effective and timely control of the disease in humans. In Italy, a serious effort has been made to strengthen the integration of human with veterinary and entomological surveillance, reaching tangible results in the prevention of the disease [3,7]. In addition, since 2014, some Italian regions have explored the feasibility of using vector and animal surveillance data to trigger blood and organ donor safety measures. Encouraging results were obtained in 2014, during a season with high level of transmission. In 2015 however, veterinary surveillance did not signal WNV circulation before the occurrence of human cases in all affected provinces. Still, when province borders are not taken strictly into account and the surrounding territories are considered, the entomological and veterinary surveillance was able to detect the virus circulation also in 2015. Veterinary surveillance identified WNV circulation in some regions (Apulia, Sardinia, Sicily) without any human cases. This could be related both to the under ascertainment or under notification of human cases to the surveillance system or to limited circulation of the virus between bird and mosquito populations in rural areas.

In conclusion, an integrated human, animal and vector surveillance is crucial to timely set up preventive measures, such as the early detection of infected blood donors.

The integration of surveillance activities and the multidisciplinary approach in Italy might be a good practice to be implemented also in other affected countries for the identification of viral circulation. They have been fundamental to implement and/or strengthen preventive measures aimed at reducing the risk of transmission of the WNV to humans.

The work that Italy has being doing in the past 8 years of surveillance is constantly enriched by an intense research activity and also by the development of innovative tools for the quantification of health risk in order to implement prevention measures and effective control.

## Notes

**Members of the WNV surveillance working group:** Francesco Maraglino, Patrizia Parodi, Corrado Cenci (Ministry of Health, Italy); Nanni Costa (National Transplant Centre, Istituto Superiore di Sanità, ISS, Italy; Giancarlo Maria Liumbruno (National Blood Centre, National Institute of Health (Istituto Superiore di Sanità, ISS, Italy); Patrizio Pezzotti, Claudia Fortuna, Cristiano Fiorentini, Maria Elena Remoli, Eleonora Benedetti, Daniela Boccolini, Marco Di Luca, Roberto Romi, Francesco Severini, Luciano Toma, Luca Busani, Silvia Declich, Gianni Rezza (Istituto Superiore di Sanità, Rome Italy); Maria Paola Landini (Regional Reference Laboratory, Emilia-Romagna); Emanuela Bedeschi, Michele Dottori, Claudio Gualanduzzi, Annalisa Lombardini, Andrea Mattivi, Silvano Natalini, Gabriele Squintani, (Emilia-Romagna Region working group for WNV surveillance); Mariarita Gismondo, Francesca Rovida, Nadia Zanchetta, Davide Rossi and Fausto Baldanti (Regional Reference Laboratories, Lombardy Region, Italy); Maria Gramegna, Marco Farioli (Regional Health Authority of Lombardy, Italy); Davide Lelli, Alice Prosperi e Francesca Faccin (Istituto Zooprofilattico Sperimentale della Lombardia e dell’Emilia-Romagna ‘Bruno Ubertini’, Brescia, Italy); Francesca Zanella (Regional Health Authority of Veneto, Italy).
